# Development and Evaluation of MCC-SiO_2_/CMC-SiO_2_ Conjugates as Tablet Super-Disintegrants

**DOI:** 10.3390/polym14051035

**Published:** 2022-03-04

**Authors:** Tanikan Sangnim, Simran Kaur Zandu, Sukhanpreet Kaur, Oluwatoyin A. Odeku, Kampanart Huanbutta, Inderbir Singh

**Affiliations:** 1 Faculty of Pharmaceutical Sciences, Burapha University, 169 Seansook, Muang District, Chonburi 20131, Thailand; tanikan@go.buu.ac.th; 2 Chitkara College of Pharmacy, Chitkara University, Rajpura 140401, Punjab, India; simran1902simi@gmail.com (S.K.Z.); sukhanpreet20010.ccp@chitkaea.edu.in (S.K.); 3 Department of Pharmaceutics and Industrial Pharmacy, Faculty of Pharmacy, University of Ibadan, Ibadan 200005, Nigeria; pejuodeku@yahoo.com; 4 School of Pharmacy, Eastern Asian University, Thanyaburi District, Pathumthani 12110, Thailand; kampanart@eau.ac.th

**Keywords:** MCC, CMC, MCC-SiO_2_ conjugates, CMC-SiO_2_ conjugates, tablet superdisintegrant

## Abstract

In the present study, microcrystallinecellulose–colloidal silicon dioxide (MCC-SiO_2_) and carboxymethylcellulose–colloidal silicon dioxide (CMC-SiO_2_) conjugates have been investigated as superdisintegrants in fast dissolving tablets (FDTs). MCC-SiO_2_ and CMC-SiO_2_ conjugates were prepared and micromeritic studies, FTIR, SEM and XRD methods were utilized for characterizing the powdered conjugates. The conjugates were used for the preparation of domperidone FDTs by direct compression and the wetting time, water absorption ratio, disintegration time and in vitro drug release were evaluated. Effective pore radius of MCC-SiO_2_ and CMC-SiO_2_ conjugates for 1:1, 1:2.5 and 1:5 was found to be 13.35 ± 0.31 µm, 15.66 ± 0.17 µm and 18.38 ± 0.44 µm, and 16.81 ± 0.24 µm, 20.12 ± 0.39 µm and 26.37 ± 0.24 µm, respectively, compared to 12.21 ± 0.23 µm for MCC and 13.65 ± 0.21 µm for CMC. The results of effective pore radius indicate the wicking capability as well as the disintegration potential of MCC-SiO_2_ and CMC-SiO_2_ conjugates over pure MCC and CMC. The results of wetting time, water absorption ratio and disintegration time for MCC-SiO_2_ conjugates were found to be in the range of 19 ± 1.21 to 30 ± 1.33 s, 42 ± 0.28 to 49 ± 0.47% and 15 ± 2 to 40 ± 1 s, and for CMC-SiO_2_ conjugates were found to be in the range of 21 ± 1.13 to 40 ± 1.17 s, 42 ± 0.94 to 49 ± 0.57% and 12 ± 2 to 20 ± 3 s, respectively. Conjugation of MCC and CMC with SiO_2_ led to the formation of a complex with remarkable tablet superdisintegrant potential that could be used in preparing fast disintegrating tablets.

## 1. Introduction

Fast disintegrating tablets (FDTs) have emerged as prominent dosage forms in the recent past, as they disintegrate rapidly within the mouth in a matter of few seconds, showing rapid dissolution and fast onset of action. Pre-gastric absorption of the FDTs in the mouth and esophagus decreases the amount of drug undergoing the first-pass metabolism and thereby results in improved bioavailability [1,2]. These dosage forms provide various other advantages such as accurate dosing, easy portability, chemical and physical stability and are highly convenient for geriatric, pediatric, bedridden and uncooperative patients [3]. They are generally represented as “quick melt”, “orally disintegrating”, “melt in mouth”, “quick dissolve”, “fast disintegrating”, “rapid melt”, “oro-dispersible”, “fast dissolve” and “mouth dissolving” etc. Disintegrants are added to solid oral dosage forms to promote the disintegration process of the compacted mass and further increase drug dissolution. Recently, newer disintegrants, known as superdisintegrants are being explored. These agents are used in low concentrations, usually 1–10% of the total weight of the dosage form when compared to other disintegrants. Various formulations employ standard super disintegrants such as croscarmellose sodium, crosspovidone and sodium starch glycolate [4].

Microcrystalline cellulose (MCC) and carboxymethyl cellulose (CMC) are remarkable excipients that are widely used as disintegrants in tablet formulations and various modifications have been done to extend their properties as tablet super disintegrants. Studies have shown that PEGylated conjugates of microcrystalline cellulose as promising super disintegrant at a concentration above 1% which led to an enhancement in water vapor uptake, water penetration rate, super-disintegration power and dissolution rate [5]. In another study, optimized mouth dissolving tablets have been prepared using a combination of glycine, carboxymethyl cellulose and sodium alginate using Plackett-Burman design and the formulation was found to decrease the disintegration and wetting times and increase the porosity and water absorption ratio [6]. Chitosan-silicon dioxide [7], starch-silicon dioxide [8], chitin-metal silicates [9] have been reported as novel tablet super-disintegrants. In the present study, MCC-SiO_2_ and CMC-SiO_2_ conjugates have been synthesized and used in the formulation of domperidone FDTs. The conjugates were evaluated by SEM, FTIR and XRD techniques and the micrometric studies were conducted to explore the flowability of the conjugates. The mechanical and drug release characteristics of domperidone FDTs were also evaluated.

## 2. Materials and Methods

### 2.1. Materials

The materials used are domperidone (gift from IPZHA Pharmaceuticals, Patiala, Punjab, India), MCC (Signet, Mumbai, India), CMC and SiO_2_ (purchased from LobaChemie, Mumbai, India) and marketed domperidone FDTs: Domstal by Prima (Torrent Pharmaceuticals Ltd., Baddi, India).

### 2.2. Preparation of MCC-SiO_2_ and CMC-SiO_2_ Conjugates

One gram of MCC/CMC dispersed in 100 mL of 2 M NaOH and one gram of SiO_2_ was dispersed in 100 mL of 2 M HCl. The silica suspension was poured into the MCC/CMC suspension with continuous stirring for an hour. The pH of the mixture was adjusted with concentrated HCl to 6.5. The resulting mixture was then transferred to a round bottom flask and 5 mL acetone was added and then freeze-dried. The freeze-dried powder was scraped off and sieved using mesh number 44 and stored in a properly sealed container. A similar procedure was used to prepare conjugate containing MCC/CMC: colloidal silicon dioxide ratios of 1:2.5 and 1:5, in which the concentration of SiO_2_ increased to 2.5 g and 5 g, respectively.

### 2.3. Characterization of MCC-SiO_2_ and CMC-SiO_2_ Conjugates

The prepared MCC-SiO_2_ and CMC-SiO_2_ conjugates were investigated for several pre-compression parameters, including micromeritic studies, loss on drying and effective pore radius. Post-compression evaluation was carried out using techniques such as FTIR, SEM and XRD.

#### 2.3.1. Micromeritic Studies

The bulk density (D_b_), tapped density (D_t_), Carr’s compressibility index (CI) Hausner’s ratio and angle of repose (θ) of the MCC-SiO_2_ and CMC-SiO_2_ conjugates were evaluated using established methods [10].

#### 2.3.2. Loss on Drying (LOD)

The presence of solvents or moisture in the conjugates was determined using the loss on drying (LOD) method. The initial weighed of the conjugate was determined (*W*_1_) and the weight of the cool sample after heating at over 100 ± 5 °C for 2 h (*W*_2_) was determined. The percentage loss was calculated as presented in Equation (1):

(1) $$\%\mathrm{LOD}=(\frac{W_{1}-W_{2}}{W_{1}})\times 100$$

#### 2.3.3. Effective Pore Radius (R_eff.p_)

The effective pore radius (*R_eff.p_*) of the powdered blend was evaluated by weighing a 2 mL micropipette tip filled with the powder (*W_i_*). Then n-hexane was added in a dropwise manner on the bed top till the solvent filtered out at the bottom of the tip and was weighed again (*W_f_*) [11]. The *R_eff.P_* was determined as described in Equation (2):

(2) $$R_{eff.p}=\frac{W_{f}-W_{i}}{2\pi y}$$

#### 2.3.4. Attenuated Total Reflectance-Fourier Transform Infrared Spectroscopy (ATR-FTIR)

The Infrared spectra of the samples were obtained using an ATR-FTIR spectrophotometer (IFS66/S, Alpha Bruker, Ettlingen, Germany). The KBr pellet technique was used to analyses the samples with spectra 4000 cm^−1^ to 400 cm^−1^. 

#### 2.3.5. X-ray Diffraction (XRD)

The X-ray diffractograms of different samples were obtained using an X-pert pro system (P analytical X’Pert Pro MRD, Malvern Panalytical Ltd., Malvern, UK) which is configured in Bragg Brentano geometry. The equipment consists of a copper anode in glass tubing and a graphite monochromator. The operating conditions were set at 40 mA and 40 kV. The powders (≤250 µm) were mounted on a glass slide arbitrarily and scanned through a range of 2θ angles.

#### 2.3.6. Scanning Electron Microscopy (SEM)

The surface morphology was examined using scanning electron microscopy (4300 SE/N SEM, Hitachi, Santa Clara, CA, USA) at an accelerating potential of 10 kV. The sample was placed on the silver plate of the specimen stage in a vacuum evaporator.

### 2.4. Preparation of Tablets

The prepared conjugates (400 mg) were compressed for 30 s using a hydraulic press (Model CAP15T-1233, PCI Analytics, Mumbai, India) to form tablet and then stored over silica gel for 24 h. The relative densities (*R*) of the tablets were determined by using the following equation:

(3) $$R=\frac{m}{V_{r}\times P_{s}}$$

where, *m* is mass, *V_r_* is the volume of tablet (cm^3^) and *P_s_* is the particle density of solid material (g/cm^3^).

#### 2.4.1. Compression Studies

Mathematical compression models are generally normalized with the inclusion of initial volume or particle density to compare the compression properties of materials. Two widely used compression models—Heckel and Kawakita—were used to study the compression properties of the composites.

#### 2.4.2. Heckel Function 

The Heckel model uses relative density to determine the compaction properties of the material. It relates the densification of the powdered material with the applied pressure. The force and displacement values (as obtained from the simulator) were determined and the apparent density of the powder bed 1/(1 − *D*) was calculated using force values (converted to pressure), displacement values and true density of the material. The compression stage was subjected to linear regression analysis using the Heckel model and is denoted by the following Equations (4) and (5):

(4) $$\ln\left(\frac{1}{1-D}\right)=KP+A$$

where, *D* is the relative density of the powdered material, *K* (slope) is the reciprocal of the mean yield pressure of the material and *P* is the applied pressure. Further, the value of *A* (intercept) can be used to calculate the relative density:

(5) $$D_{A}\times D_{A}=1-e^{-A}D_{B}=D_{A}-D_{0}$$

where, *D_B_* is the relative density at low pressures and *D_0_* is the relative density when no pressure is applied [12,13].

#### 2.4.3. Kawakita Function 

The Kawakita model uses the relative volume change to determine the compressibility of pharmaceutical powders. It is calculated using the following formula:

(6) $$C=\frac{V_{0}-V_{p}}{V_{p}}=\frac{abp}{1+bp}$$

where, *C* is the degree of volume reduction, *V_0_* is the initial bulk volume of the powder, *V_p_* is the bulk volume under pressure, ‘*a*’ is a constant related to minimum porosity of material before compression and ‘*b*’ is a constant associated with the plasticity of the material [12,13]. The simplified equation, in practice, is given by:

(7) $$\frac{P}{C}=\frac{P}{a}+\frac{1}{ab}$$

### 2.5. Preparation of Fast Disintegrating Tablets

Direct compression technique was used to prepare FDTs of domperidone. Table 1 incorporating MCC, MCC-SiO_2_ conjugates and CMC, CMC-SiO_2_ conjugates as superdisintegrant were prepared in three different ratios viz. 1:1, 1:2.5 and 1:5 according to the formulae. Fast disintegrating tablets each containing 10 mg of the drug were prepared and Avicel 102 was used as a diluent. The amount of drug, diluent and MCC/MCC-SiO_2_ conjugates or CMC/CMC-SiO_2_ conjugates were carefully weighed, sieved through the mesh of size 40 and finally mixed for 15–20 min using the tumbling mixer. To the mixture, talc and magnesium stearate were added as lubricants and then compressed into tablets using a multi-punch tableting machine (AK Industries, Nakodar, Punjab, India).

#### 2.5.1. Wetting Time

A culture dish was filled with a 6 mL solution of Eosin (water-soluble dye) and twice-folded tissue paper (10.75 m × 12 m). The tablet was kept on top of the tissue paper and the wetting time was recorded as the time which the tablets took to absorb the solution to its top surface [14].

#### 2.5.2. Water Absorption Ratio

The water absorption ratio was determined by a method similar to the wetting time technique. The tablet was weighed before and after complete wetting took place. The following formula was used for calculating the water absorption ratio:

(8) $$R=\left(\frac{W_{b}-W_{a}}{W_{a}}\right)\times 100$$

where *W_a_* refers to the weight of tablet before absorption of water and *W_b_* refers tothe weight of tablet after absorption of water [15].

#### 2.5.3. In Vitro Disintegration Time

The disintegration test for the fast disintegrating tablets was conducted using USP disintegration apparatus (EI Product, Panchkula, India) in 900 mL of 0.1N HCl.

#### 2.5.4. In Vitro Dissolution Study

The in vitro dissolution was investigated using the USP dissolution apparatus II (Paddle type -Lab India DS 8000, Mumbai, India). For the study, 0.1 N HCL (900 mL, 37 ± 0.5 °C) was used as dissolution medium and the paddles was rotated at the speed of 50 revolutions per min. Sampling was done by drawing out 5 mL samples after particular periods and filtering them using Whatman filter paper. The samples were then appropriately diluted and analyzed with the help of a UV-Visible spectrophotometer (Systronics, Mumbai, India) at λ_max_ 284 nm. The drug concentration was determined and expressed as cumulative percent drug released. The similarity factor (*f*2) is considered to be the logarithmic reciprocal square root transformation of the sum-squared error. It is a convenient way of comparing the dissolution rates if the dissolution time points are greater than three or four. The similarity factor is calculated as presented Equation (9):

(9) $$f2=50\times \log\left\{\left[1+\left(\frac{1}{n}\right)\sum_{j=1}^{n}W_{j}|R_{j}-T_{j}|2\right]-0.5\times 100\right\}$$

where *W_j_* is an optional weight factor, *R_j_* is the percentage of reference sample dissolved at time t and *T_j_* is the percentage of test sample dissolved at time t. The *f*2 value rangefrom 0 to 100. If the *f*2 value is 100 then both the test as well as reference samples are identical whereas it tends to be 0 if they are non-identical. The *f*2 values should be nearer to 100, to depict a similar dissolution profile.

#### 2.5.5. Friability Test

The friability (F) of 20 tablet were measured using a Rocha friabilator (Campbell Electronics, Mumbai, India). As per USP 30-NF 25, tablets were weighed and then rotate at 25 rpm for 4 min. Tablet were taken out, dedusted and reweighed. The limit of the friability test shall not be more than 1.0%

#### 2.5.6. Hardness

The tablet was placed in a vertically holding edges of the anvil of a Monsanto Hardness Tester (Model VMT-1, VinSyst Technologies, Mumbai, India). The pointer is adjusted at zero position on the scale, then the screw rotated till the break point of the tester. The breakage of tablet shows hardness on the scale. The test was performed on six tablets and the average was calculated.

#### 2.5.7. Tensile Strength

Tensile strength of the tablet is the force required to break the tablet by compression in the radial direction or it is measure of stress necessary to Couse diametric compaction of the compact and it is measure by a Monsanto Hardness Tester. For measuring the hardness of tablet, the plunger of the hardness tester is driven down at the speed of 20 mm/min. Tensile strength were calculated by following formula:

T = 2F/πDt(10)

where F is the crushing load, D diameter and t thickness of tablet.

## 3. Results

### 3.1. Pre-Compression Evaluation

The micromeritic properties of the conjugates are presented in Table 2 and Table 3. The studies primarily included evaluation of bulk density, tapped density, Carr’s compressibility index, Hausner’s ratio, angle of repose, loss on drying and effective pore radius. The flow properties of MCC and CMC were improved with the addition of SiO_2_. This can be confirmed from the angle of repose values; 34.52–29.19° for MCC-SiO_2_ conjugates and 33.24–28.89° for CMC-SiO_2_ conjugates when compared to that of MCC (35.66°) and CMC (36.56°). Further, Carr’s index values were found to be in the range of 19.42 to 11.52 for MCC-SiO_2_ conjugates and 16.77 to 12.19 for CMC-SiO_2_ conjugates, depicting good to fair flow rate when compared to MCC (20.17) and CMC (19.43). Hausner’s ratio ranged from 1.24 to 1.13 for MCC-SiO_2_ conjugates and 1.20 to 1.14 for CMC-SiO_2_ conjugates, indicating enhanced flowability in comparison to MCC (1.25) and CMC (1.24).

Loss on drying for MCC-SiO_2_ conjugates was found to be 9.53 ± 0.20% (1:1), 9.29 ± 0.37% (1:2.5) and 9.11 ± 0.46% (1:5), and for CMC-SiO_2_ conjugates was found to be 9.60 ± 0.12% (1:1), 9.45 ± 0.26% (1:2.5) and 9.28 ± 0.22% (1:5) when compared to 11.10 ± 0.15% for MCC and 9.88 ± 0.09% for CMC. Effective pore radius of MCC-SiO_2_ and CMC-SiO_2_ conjugates for 1:1, 1:2.5 and 1:5 was found to be 13.35 ± 0.31 µm, 15.66 ± 1.17 µm and 18.38 ± 0.44 µm, and 16.81 ± 0.24 µm, 20.12 ± 0.39 µm and 26.37 ± 0.24 µm, respectively, when compared to 12.21 ± 1.23 µm for MCC and 13.65 ± 0.21 µm for CMC. The results of effective pore radius demonstrate the wicking capability as well as the disintegration potential of MCC-SiO_2_ and CMC-SiO_2_ conjugates over pure MCC and CMC. The results of micromeritic study indicates that amongst the three ratios of MCC-SiO_2_ and CMC-SiO_2_ conjugates, 1:5 was the most effective ratio followed by 1:2.5 and 1:1 [16].

### 3.2. Instrumental Evaluation

#### 3.2.1. ATR-FTIR Analysis

**Figure 1:**
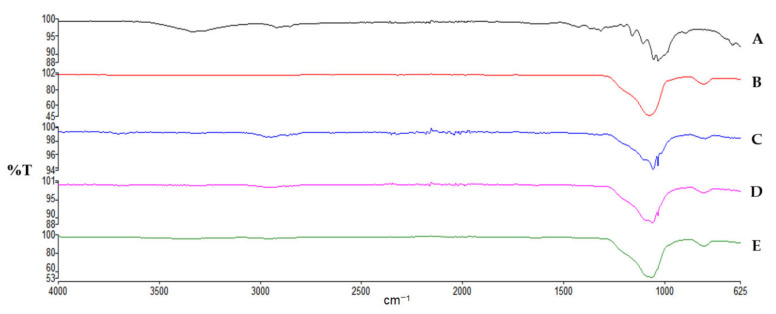
IR spectra of (A) MCC, (B) SiO_2_ and MCC-SiO_2_ conjugates prepared in ratio; (C) 1:1, (D) 1:2.5 and (E) 1:5.

**Figure 2:**
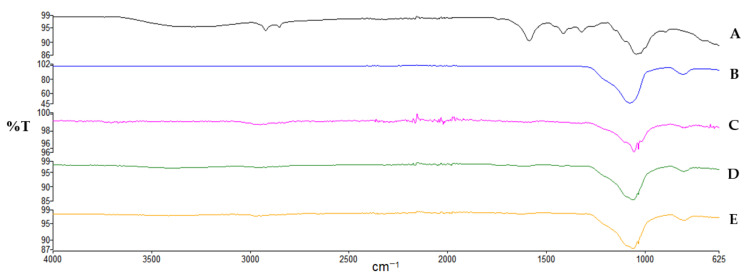
IR spectra of (A) CMC, (B) SiO_2_ and CMC-SiO_2_ conjugates prepared in ratio; (C) 1:1, (D) 1:2.5 and (E) 1:5.

Analysis of the spectrum of microcrystalline cellulose (MCC) shown in Figure 1. revealed the existence of a wide band in the 3336 cm^−1^ region signifying a large number of hydrogen bonds, formed by-OH groups contributing to the polymer’s stiffness and tight chain packing. In the 2913 cm^−1^ region the band corresponding to the symmetric and asymmetric vibrations of the CH_2_ group is observed. The 1440–1255 cm^−1^ range indicates the deformation vibrations of CH_2_ and CH groups as well as the angular deformation vibrations of C-O-H. The peak at 1032 cm^−1^ represents C-O-C bending vibrations. The MCC-SiO_2_ conjugates exhibited a sharp peak near 1076 cm^−1^, which indicates a strong interaction between the surface OH groups of cellulose and silica particles, with the emergence of Si-O-C bridging bond. With an increase in the concentration of SiO_2_, this sharp peak transforms into a broad band. Further, a decrease in the intensity of the peaks at 3336 cm^−1^ and 2913 cm^−1^ validates the presence of intermolecular bonding between silicon dioxide and MCC, which could be responsible for the increased disintegration behavior of MCC-SiO_2_ conjugates. The IR spectra of CMC shown in Figure 2 revealed a wide indistinct band at 3200–3400 cm^−1^ representing –OH stretching and a sharp peak at 2921.40 cm^−1^ which represents the symmetric and asymmetric vibrations of –CH_2_ groups. The peaks at 1586.04 cm^−1^ and 1414.8 cm^−1^ represent the asymmetric and symmetric stretching of the carboxylate group, respectively. Further, the peak at 1323.5 cm^−1^ represents –CH_2_ scissoring and the one at 1044.42 cm^−1^ represents C-O-C vibrations. The CMC-SiO_2_ conjugates exhibit a decrease in the intensity of all the peaks. The sharpening of the peak at 1044 cm^−1^ indicates the formation of the Si-O-C bridge between the –OH group of CMC and silicon of SiO_2_. This interaction can be held responsible for the reduction in the time for tablet disintegration.

#### 3.2.2. XRD Analysis

**Figure 3:**
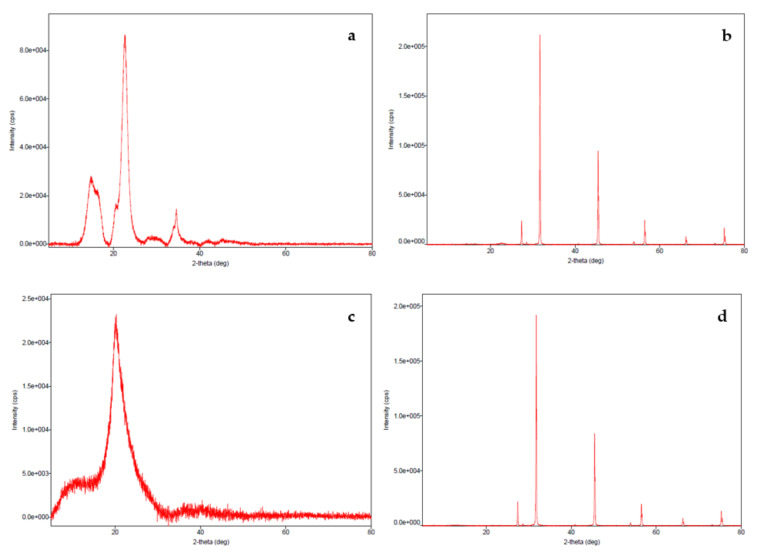
X-ray diffraction pattern of (**a**) MCC, (**b**) MCC-SiO_2_ conjugates, (**c**) CMC and (**d**) CMC-SiO_2_ conjugates.

The XRD patterns for MCC and MCC- SiO_2_ conjugates are depicted in Figure 3. The presence of broad peaks at 14.99°2θ, 20.77°2θ, 22.61°2θ, 30.30°2θ and 34.70°2θ angle indicates the structure of MCC. The appearance of relatively sharp peaks at 22.53°2θ, 27.36°2θ, 31.71°2θ, 45.45°2θ and 56.47°2θ angles in MCC-SiO_2_ conjugates corresponds to the increase in crystallinity after modification. Chemical treatment of MCC with SiO_2_ for developing the conjugates leads to the enhancement in crystallinity of the conjugates. This depicts an increase in the superdisintegrant activity of the conjugates. The structure of CMC is identified by the presence of broad peak at 20.10°2θ angle. Further, the sharp peaks at 28.45°2θ, 33.78°2θ, 44.53°2θ, 56.55°2θ and 67.04°2θ angles in the case of conjugates indicate an increase in the crystalline nature. This results in enhanced water holding capacity, thereby potentiating the use of the conjugates as tablet super disintegrant.

#### 3.2.3. SEM Analysis

**Figure 4:**
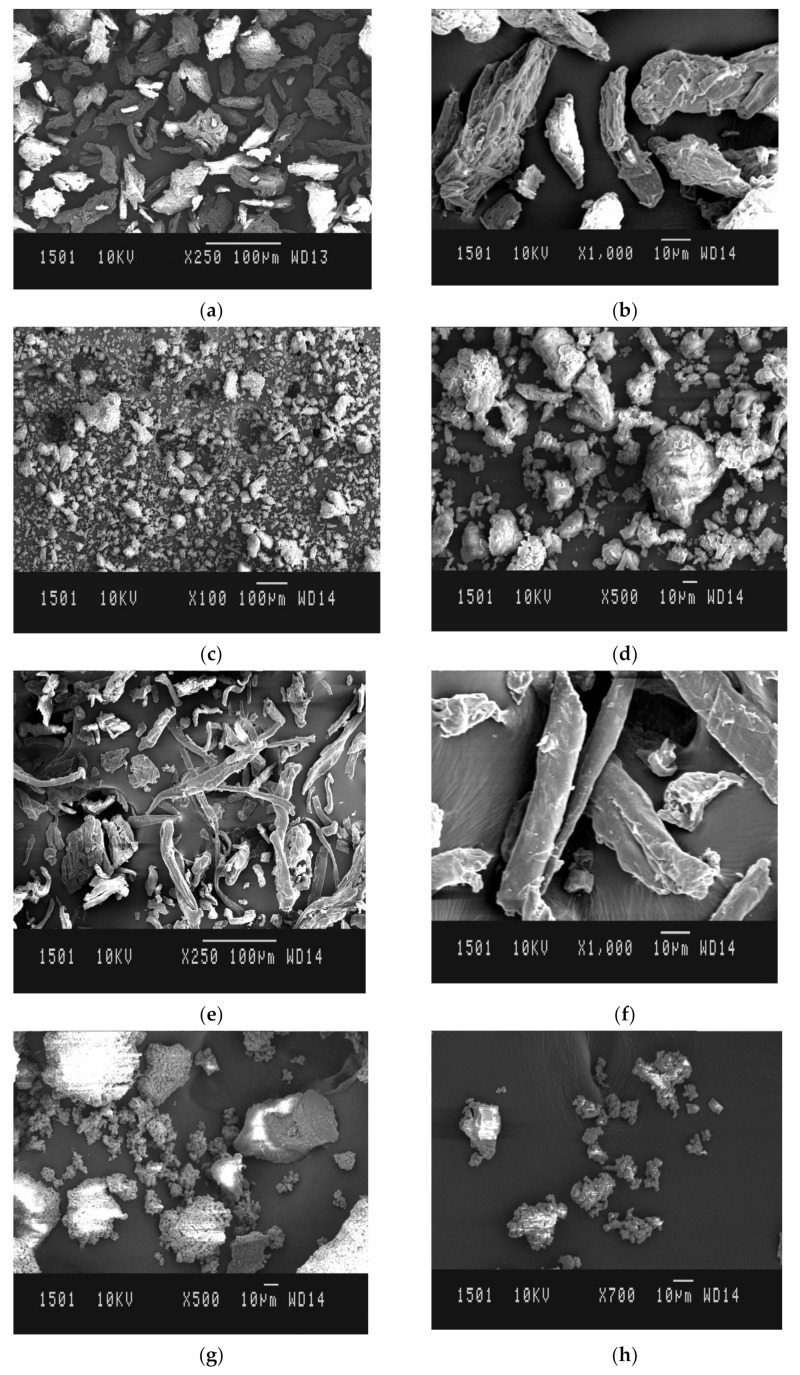
SEM photomicrograph of (**a**,**b**) MCC, (**c**,**d**) MCC-SiO_2_ conjugates prepared in the ratio 1:5, (**e**,**f**) CMC, and (**g**,**h**) CMC-SiO_2_ conjugates prepared in the ratio 1:5.

The SEM micrographs of MCC (Figure 4a,b) showed discrete and irregularly shaped structures. Cellulose microfibrils with unequal distribution are also evident in the images. Conjugation of MCC with SiO_2_ damages the microfibrillar structure of cellulose moiety into small fragments, which enhances the total surface area of the conjugates. MCC shows a non-porous structural appearance whereas MCC-SiO_2_ conjugates (Figure 4c,d) had a more porous structure. Increased surface area and pores in the surface morphology of MCC-SiO_2_ conjugates could be held responsible for their improved tablet super disintegrant property.

The SEM micrographs of CMC (Figure 4e,f) showed tubular rod shaped microfibrils of cellulose, having smooth surface morphology. The conjugates (Figure 4g,h) on the other hand showed fragmentation of cellulose microtubules into tiny particles, thereby increasing the surface area. The presence of inter-particle voids in the prepared CMC-SiO_2_ conjugates as depicted by the SEM micrographs could be the reason for the wicking action, which enhanced water uptake of the conjugates.

### 3.3. Compression Study

#### 3.3.1. Heckel Function Analysis

The D_o_ value, which is the extent of packing when the powder is being filled in the die for MCC was found to be 0.791 and it reduced from 0.786 to 0.569 for MCC: SiO_2_ conjugates in the ratios of 1:1 to 1:5, respectively. Generally, increased porosity of powders is related to lower values of D_o_. In addition, the D_B_ value represents the densification of powder bed at low pressure, further reflecting particle rearrangement and fragmentation (plastic/elastic) when pressure is applied. The MCC conjugates prepared in the ratio 1:5 exhibited the highest D_B_ value (0.224) while pure MCC exhibited the lowest value (0.126). This demonstrates that more fragmentation takes place within the 1:5 conjugates when compared to the other ratios. D_A_ exhibits the overall degree of densification at zero and low pressures. It was found to be in the order of 1:1 > 1:2.5 > 1:5 for MCC-SiO_2_ conjugates, thereby indicating the highest degree of packing in 1:1conjugates. Further, P_y_ is the mean yield pressure and is inversely proportional to the plastically deforming ability of the material which undergoes pressure. The results conclude that conjugates in the ratio of 1:5 depict the fastest onset of plastic deformation whereas the conjugates in the ratio of 1:1 depict the slowest onset. 

**Figure 5:**
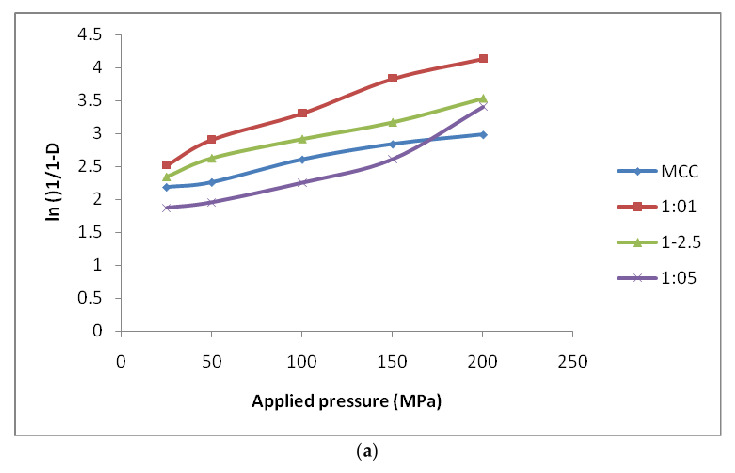

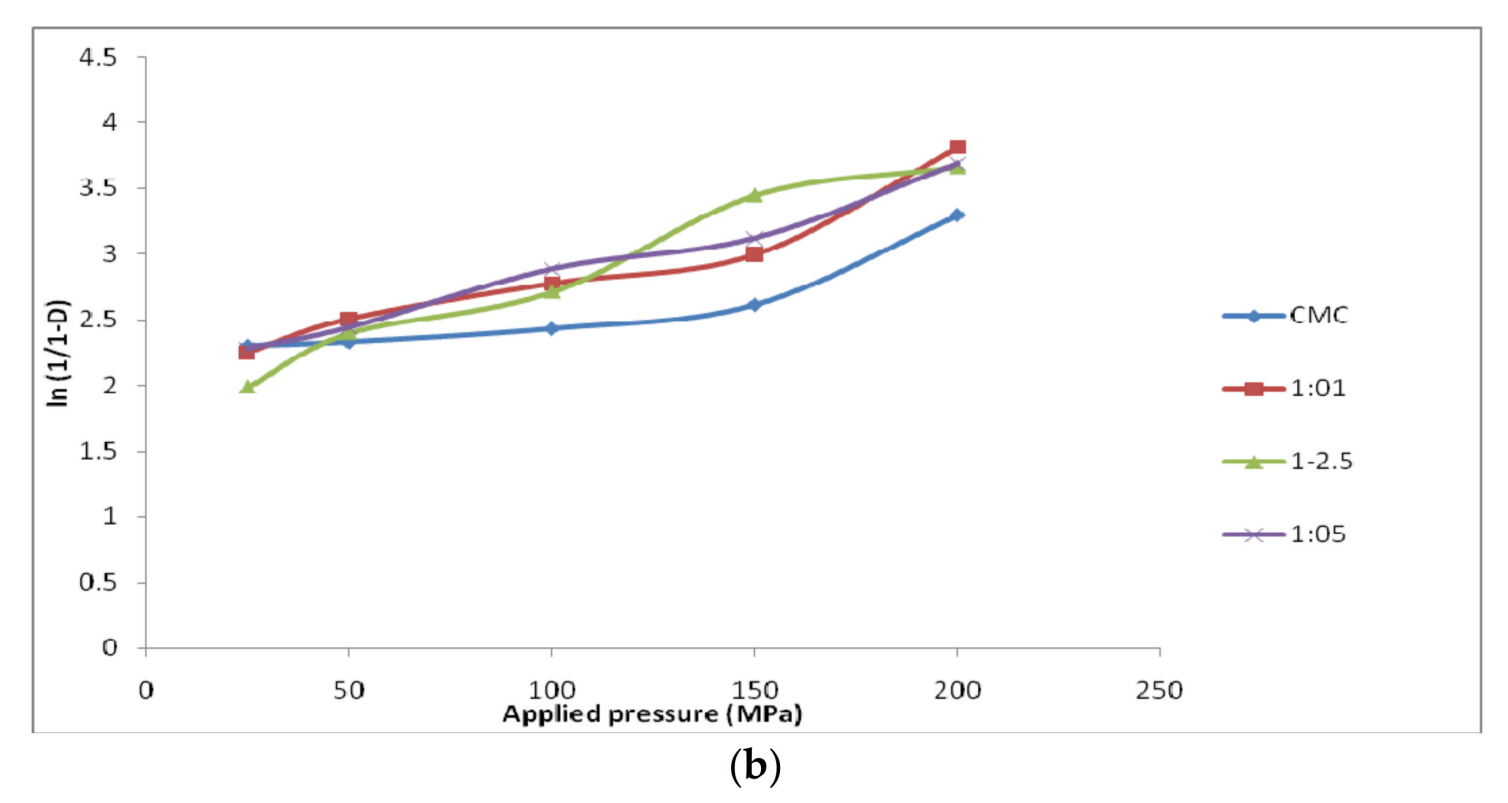
Heckel- Plots for the tablet incorporating (**a**) MCC-SiO_2_ conjugates as a super-disintegrant prepared in ratio 1:1, 1:2.5 and 1:5. (**b**) CMC-SiO_2_ conjugates as a superdisintegrant prepared in ratio 1:1, 1:2.5 and 1:5.

Figure 5 show representative Heckel plots for MCC and MCC-SiO_2_ conjugates, and CMC and CMC-SiO_2_ conjugates, respectively, prepared in different ratios, 1:1, 1:2.5 and 1:5. The D_o_ of CMC was found to be 0.772 and its value reduces from 0.748 to 0.679 for MCC-SiO_2_ ratios of 1:1 to 1:5, respectively. The CMC conjugates prepared in the ratio 1:5 exhibited the highest D_B_ value (0.203) while pure MCC exhibited the lowest value (0.101). This demonstrates a higher degree of fragmentation taking place in the 1:5 conjugates. The values of D_A_ were in the rank order of 1:1 > 1:2.5 > 1:5 for CMC-SiO_2_ conjugates, with a 1:1 ratio showing a higher degree of packing at low pressures. Further, in terms of mean yield pressure (P_y),_ the conjugates of 1:5 exhibit the fastest onset of plastic deformation whereas the conjugates of 1:1 exhibit the slowest onset [17,18]. 

#### 3.3.2. Kawakita Function Analysis

**Figure 6:**
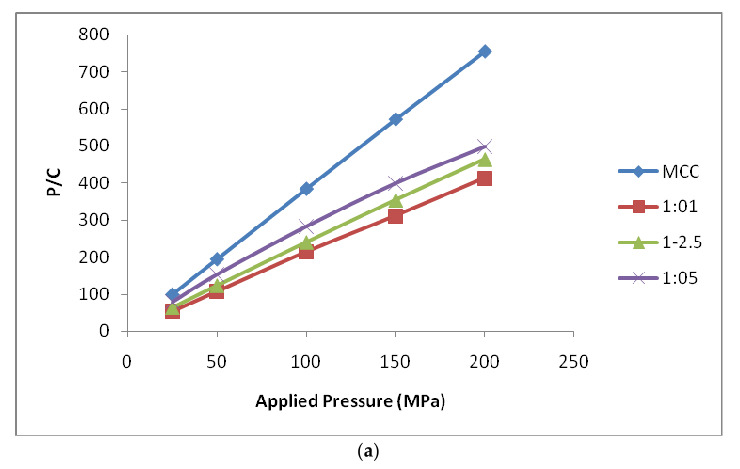

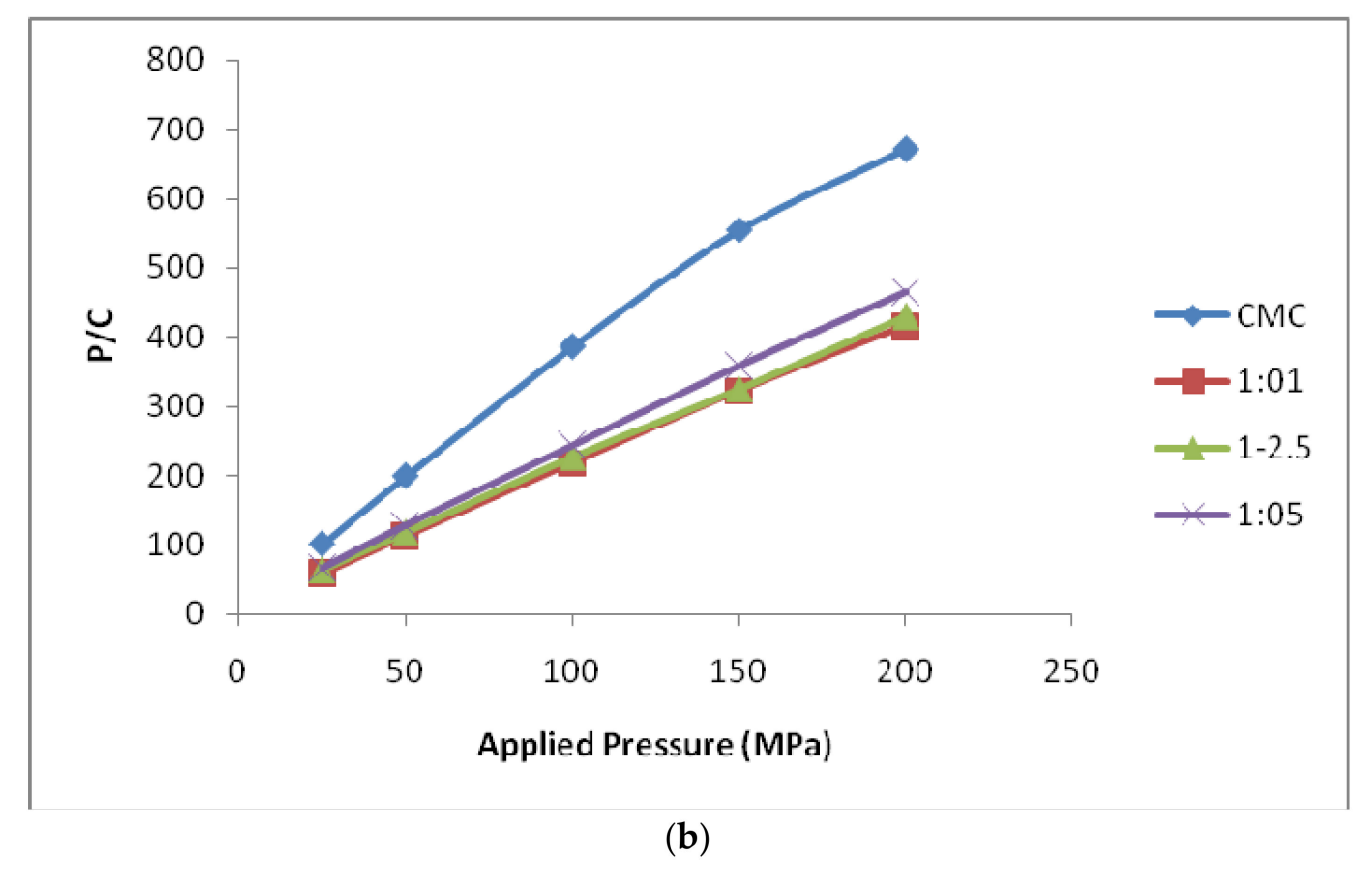
Kawakita- Plots for the tablet incorporating (**a**) MCC-SiO_2_ conjugates as a super-disintegrant prepared in ratio 1:1, 1:2.5 and 1:5. (**b**) CMC-SiO_2_ conjugates as a superdisintegrant prepared in ratio 1:1, 1:2.5 and 1:5.

The Kawakita plots for the MCC and MCC-SiO_2_ conjugates, and CMC and CMC-SiO_2_ conjugates prepared in different ratios 1:1, 1:2.5 and 1:5 presented in Figure 6. The results of D_I_ and P_k_ are listed in Table 4 and Table 5. The D_I_ value was 0.733 for MCC and 0.510, 0.562, 0.582 for MCC-SiO_2_ conjugates of ratio 1:1, 1:2.5, 1:5, respectively. The value of P_k_ is inversely related to the extent of plastic deformation taking place during the compression process and was observed to be 1.789 for MCC and 2.758, 3.995 and 5.141 for MCC-SiO_2_ conjugates of ratio 1:1, 1:2.5, 1:5, respectively. Therefore, the ability of material to undergo plastic deformation during compression could be rated as MCC > MCC-SiO_2_; 1:1 > MCC-SiO_2_; 1:2.5 > MCC-SiO_2_; 1:5. Further, the value of 𝑃_𝑦_ indicates the onset of plastic deformation during the compression process.

The D_I_ was 0.697 for CMC and 0.513, 0.521, 0.562 for CMC-SiO_2_ conjugates of ratio 1:1, 1:2.5, 1:5, respectively. The value of P_k_ was found to be 2.541, 4.519, 6.122 and 6.904 for CMC and CMC-SiO_2_ conjugates of ratio 1:1, 1:2.5, 1:5, respectively. The ability of material to undergo plastic deformation during compression could be rated as CMC > CMC-SiO_2_; 1:1 > CMC-SiO_2_; 1:2.5 > CMC-SiO_2_; 1:5 [19,20].

### 3.4. Post-Compression Evaluation

#### 3.4.1. Size, Friability, Hardness and Tensile Strength of the Formulated FDTs

All the fast disintegrating tablets were prepared under identical circumstances to prevent any variation in the formulation process. The diameter varied from 6.72 to 6.73 mm for all the formulations of MCC-SiO_2_ tablets and 6.73 to 6.74 mm for all the formulations of CMC-SiO_2_ tablets. The thickness varied from 3.03 to 3.04 mm for all the samples of MCC and CMC. Further, the hardness of MCC-SiO_2_ tablets and CMC-SiO_2_ tablets was found to be in the range of 3.11–2.90 Kg/cm^2^, and 3.30–3.01 Kg/cm^2^, respectively, as compared to 2.65 and 2.95 Kg/cm^2^ of tablets formulated using pure MCC and pure CMC, respectively. The percentage friability of all the samples was found to be less than 1%, thereby demonstrating sufficient physical properties. Tensile strength was observed to be high in case of MCC-SiO_2_ and CMC-SiO_2_ when compared to that of pure MCC and pure CMC. This can be attributed to the fact that addition of SiO_2_ forces the particles to come closer together during compression, resulting in tighter packing and further increasing the tensile strength of the tablets. Table 6 lists the parameters including diameter, thickness, friability, hardness and tensile strength of the formulated FDTs.

#### 3.4.2. Wetting Time, Water Absorption Ratio, Disintegration Time and Drug Content of the Prepared FDTs

Table 7 lists various values depicting the wetting time, water absorption ratio, disintegration time and drug content of the prepared FDTs. Wetting time was found to be 35 s for the tablets incorporating pure MCC; it decreased from 30 to 19 s as the ratio of MCC-SiO_2_ conjugates increased from 1:1 to 1:5. In addition, disintegration time reduced from 40 s for the tablets constituting MCC to 23, 18 and 15 s for the tablets constituting MCC-SiO_2_ conjugates in the ratios 1:1, 1:2.5 and 1:5. Similarly, wetting time and disintegration time were observed to be less in case of tablets constituting CMC-SiO_2_ conjugates when compared to tablets constituting pure CMC. Further, a decrease in time was observed with increase in the ratio of the CMC-SiO_2_ conjugates, i.e., 1:1 > 1:2.5 > 1:5. Water absorption ratio was observed to be inversely related to the wetting and disintegrating time of the tablets of all batches. The fast disintegration of tablets comprising the conjugates can be attributed to the formation of voids which promote quick penetration of the dissolution medium into the tablets. This further leads to swelling and wicking action, creating hydrodynamic pressure inside the tablets and destroying the physical bonds between the particles [21]. 

#### 3.4.3. In Vitro Dissolution Studies and Similarity Factor (*f*2)

**Figure 7:**
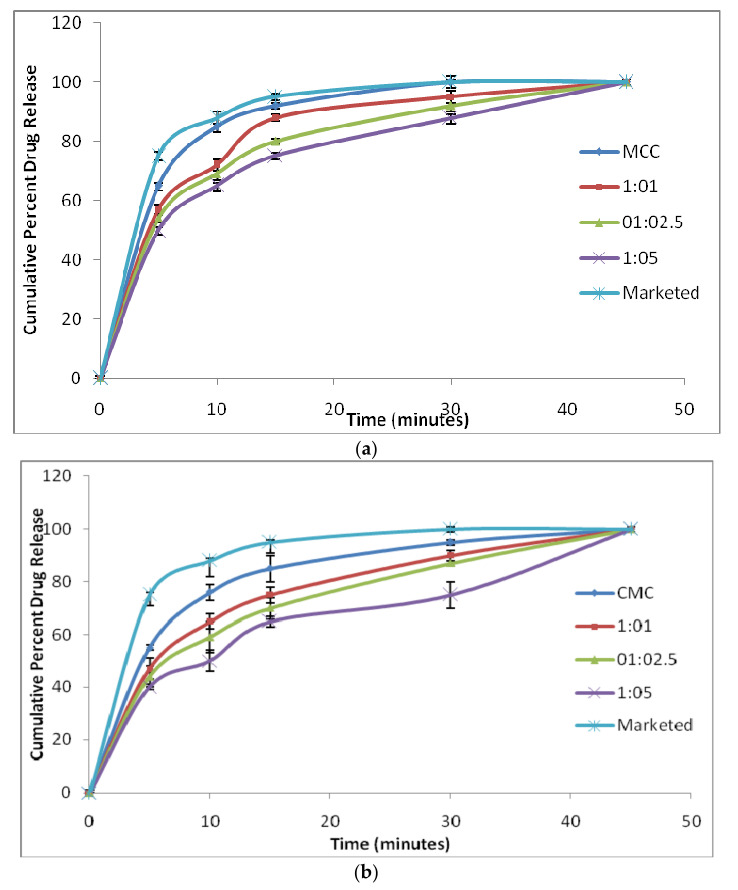
In vitro drug release profiles of (**a**) MCC and MCC-SiO_2_ conjugates FDTsand (**b**) CMC and CMC-SiO_2_ conjugates FDTs.

The formulated FDTs were compared with the marketed formulation of Domperidone (MKTD). Figure 7 depicts the in vitro dissolution results. The order of drug dissolution for the different ratios of conjugates was found to be in the order of 1:1 > 1:2.5 > 1:5. For the FDTs prepared by using the conjugates, the percentage cumulative drug release was observed to decrease with increasing concentration of SiO_2_. This was due to the fact that SiO_2_ acts as a viscosifying agent. It forms a viscous layer around the drug, which in turn decreased the rate of drug dissolution. The *f*2 values obtained from the in vitro dissolution studies of the prepared FDTs are mentioned in Table 8.

#### 3.4.4. Stability Study

The formulated tablets were assessed for their stability by storing them at temperature and relative humidity of 40 ± 2 °C and 75% RH, respectively, for time intervals of 0 day, 1, 2 and 3 months. The samples were characterized for parameters including, hardness, friability, drug content and disintegration time. As evident from Table 9 and Table 10, the formulations containing the polymers in ratio 1:5 exhibited the best results.

## 4. Conclusions

The present study entailed the development of MCC-SiO_2_ and CMC-SiO_2_ conjugates in the ratio of 1:1, 1:2.5, and 1:5, with an objective of using them as tablet superdisintegrants. Various characterization tests including micromeritic studies, effective pore radius and loss on drying were performed on the powder samples. All the samples containing conjugates possessed good powder flowability. The effective pore radius of pure MCC and CMC were found to be 12.21 ± 0.23 µm and 13.65 ± 0.21 µm, respectively, whereas the MCC-SiO_2_ and CMC-SiO_2_ conjugates developed in the ratio 1:1, 1:2.5 and 1:5 showed effective pore radius 13.35 ± 0.31 µm, 15.66 ± 0.17 µm and 18.38 ± 0.44 µm, and 16.81 ± 0.24 µm, 20.12 ± 0.39 µm and 26.37 ± 0.24 µm, respectively. ATR-FTIR, XRD and SEM were used to characterize the conjugates. FTIR spectra showed intermolecular bridging in MCC-SiO_2_ conjugates as well as CMC-SiO_2_ conjugates, contributing to be the major reason for faster disintegration of the conjugates. SEM micrographs revealed that the fragmentation of cellulose microtubules into tiny particles along with the interparticulate spaces potentiated the wicking action as well as the superdisintegrant property of the conjugates. Additionally, compression studies, based on the Heckel and Kawakita methods were carried out for the formulated MCC-SiO_2_ and CMC-SiO_2_ conjugates. FDTs containing domperidone were formulated utilizing pure samples (MCC and CMC) and their conjugates (MCC-SiO_2_ and CMC-SiO_2_). The tablets were assessed for their physical properties, in vitro disintegration time, water absorption ratio and wetting time. Stability studies showed no alteration in the appearance as well as the performance of the formulations.

## Figures and Tables

**Table 1:** Formula of fast disintegrating tablets of MCC and MCC-SiO_2_ conjugates and formula of fast disintegrating tablets of CMC and CMC-SiO_2_ conjugates.

Formula of Fast Disintegrating Tablets of MCC and MCC-SiO_2_ Conjugates									
Code	Ingredients (mg)								
	Domperidone	Native CMC	CMC-SiO_2_ Conjugate			Avicel 102	Magnesium Stearate	Talc	TW *
			1:1	1:2.5	1:5				
F1	10	10	-	-	-	78	1	1	100
F2	10	-	10	-	-	78	1	1	100
F3	10	-	-	10	-	78	1	1	100
F4	10	-	-	-	10	78	1	1	100
**Formula of Fast Disintegrating Tablets of CMC and CMC-SiO_2_ Conjugates**									
**Code**	**Ingredients (mg)**								
	**Domperidone**	**Native MCC**	**MCC-SiO_2_ Conjugate**			**Avicel 102**	**Magnesium Stearate**	**Talc**	TW *
			**1:1**	**1:2.5**	**1:5**				
F1	10	10	-	-	-	78	1	1	100
F2	10	-	10	-	-	78	1	1	100
F3	10	-	-	10	-	78	1	1	100
F4	10	-	-	-	10	78	1	1	100

TW * = Total Weight of the tablet (mg).

**Table 2:** Micromeritic properties for MCC and MCC-SiO_2_ conjugates.

Parameter	Observation			
	MCC	MCC-SiO_2_ Conjugate		
		1:1	1:2.5	1:5
Bulk density (g/cm^3^)	0.368	0.581	0.532	0.476
Tapped density (g/cm^3^)	0.461	0.721	0.628	0.538
Carr’s index (%)	20.17	19.42	15.29	11.52
Hausner ratio	1.25	1.24	1.18	1.13
Angle of repose (°)	35.66	34.52	32.64	29.19
LOD (%)	11.10 ± 0.15	9.53 ± 0.20	9.29 ± 0.37	9.11 ± 0.46
Effective pore radius (µm)	12.21 ± 1.23	13.35 ± 0.31	15.66 ± 1.17	18.38 ± 0.44

**Table 3:** Micromeritic study result for CMC and CMC-SiO_2_ conjugates.

Parameter	Observation			
	CMC	CMC-SiO_2_ Conjugate		
		1:1	1:2.5	1:5
Bulk density (g/cm^3^)	0.568	0.556	0.506	0.504
Tapped density (g/cm^3^)	0.705	0.668	0.602	0.574
Carr’s index (%)	19.43	16.77	15.95	12.19
Hausner ratio	1.24	1.20	1.19	1.14
Angle of repose (°)	36.56	33.24	31.66	28.89
LOD (%)	9.88 ± 0.09	9.60 ± 0.12	9.45 ± 0.26	9.28 ± 0.22
Effective pore radius (µm)	13.65 ± 0.21	16.81 ± 0.24	20.12 ± 0.39	26.37 ± 0.24

**Table 4:** Parameters involved in Heckel and Kawakita analysis for tablet constituting MCC-SiO_2_ conjugates in the ratios 1:1, 1:2.5 and 1:5.

	Heckel Analysis				Kawakita Analysis	
	D_0_	D_A_	D_B_	P_y_	D_I_	P_k_
MCC	0.791	0.916	0.126	68.54	0.733	1.789
1:1	0.786	0.917	0.137	62.10	0.510	2.758
1:2.5	0.750	0.894	0.144	56.33	0.562	3.995
1:5	0.569	0.784	0.224	51.85	0.582	5.141

**Table 5:** Parameters involved in Heckel and Kawakita analysis for tablet constituting CMC-SiO_2_ conjugates in the ratios 1:1, 1:2.5 and 1:5.

	Heckel Analysis				Kawakita Analysis	
	D_0_	D_A_	D_B_	P_y_	D_I_	P_k_
CMC	0.772	0.871	0.101	75.11	0.697	2.541
1:1	0.748	0.867	0.127	67.84	0.513	4.519
1:2.5	0.760	0.838	0.138	60.96	0.521	6.122
1:5	0.679	0.833	0.203	57.63	0.562	6.904

**Table 6:** Values depicting the size, friability, hardness and tensile strength of MCC and prepared MCC-SiO_2_ FDTs in the ratio of 1:1, 1:2.5 and 1:5, Values depicting the size, friability, hardness and tensile strength of CMC and prepared CMC-SiO_2_ FDTs in the ratio of 1:1, 1:2.5 and 1:5.

Formulation Code	Diameter (mm)	Thickness (mm)	Friability (%)	Hardness (Kg/cm^2^)	Tensile Strength (MN/m^2^)
MCC	6.73 ± 0.02	3.03 ± 0.01	0.80 ± 0.02	2.65 ± 0.01	0.827 ± 0.015
1:1	6.73 ± 0.03	3.03 ± 0.03	0.64 ± 0.01	2.90 ± 0.05	0.905 ± 0.005
1:2.5	6.72 ± 0.01	3.04 ± 0.04	0.59 ± 0.03	3.09 ± 0.09	0.963 ± 0.018
1:5	6.72 ± 0.04	3.04 ± 0.04	0.56 ± 0.02	3.11 ± 0.15	0.969 ± 0.011
CMC	6.73 ± 0.01	3.04 ± 0.01	0.86 ± 0.02	2.95 ± 0.08	0.887 ± 0.011
1:1	6.74 ± 0.02	3.04 ± 0.03	0.80 ± 0.03	3.01 ± 0.03	0.935 ± 0.001
1:2.5	6.73 ± 0.02	3.03 ± 0.02	0.73 ± 0.04	3.20 ± 0.14	0.999 ± 0.014
1:5	6.74 ± 0.02	3.03 ± 0.03	0.67 ± 0.02	3.30 ± 0.05	1.029 ± 0.020

**Table 7:** Values depicting the wetting time, water absorption ratio, disintegration time and drug content of the prepared MCC-SiO_2_ FDTs, Values depicting the wetting time, water absorption ratio, disintegration time and drug content of the prepared CMC-SiO_2_ FDTs.

Formulation Code	Wetting Time (s)	Water Absorption Ratio (%)	Disintegration Time (s)	Drug Content (%)
MCC	35 ± 1.15	38 ± 0.40	40 ± 1	97.15 ± 0.3
1:1	30 ± 1.33	42 ± 0.28	23 ± 2	97.82 ± 0.2
1:2.5	24 ± 1.56	46 ± 0.56	18 ± 3	98.75 ± 1.0
1:5	19 ± 1.21	49 ± 0.47	15 ± 2	99.23 ± 0.7
CMC	45 ± 1.21	39 ± 0.77	43 ± 2	97.48 ± 0.8
1:1	40 ± 1.17	42 ± 0.94	20 ± 3	98.66 ± 0.3
1:2.5	30 ± 1.33	45 ± 0.08	18 ± 1	98.89 ± 1.0
1:5	21 ± 1.13	49 ± 0.57	12 ± 2	99.35 ± 0.2

**Table 8:** Values depicting *f*2 values obtained from the in vitro dissolution studies of the formulated MCC-SiO_2_ FDTs and CMC-SiO_2_ FDTs.

Formulation Code	*f*2 Value	Formulation Code	*f*2 Value
MCC	70	CMC	52
1:1	52	1:1	41
1:2.5	47	1:2.5	37
1:5	42	1:5	32

**Table 9:** Stability study for the prepared MCC-SiO_2_ FDTs.

Formulation Code	Time Interval (Months)	Test Parameters (MCC-SiO_2_)			
		Hardness (kg/cm^2^)	Friability (%)	Drug Content (%)	Disintegration Time (s)
MCC	0	3.10 ± 0.02	0.80 ± 0.02	97.15 ± 0.3	40 ± 1
	1	3.09 ± 0.15	0.81 ± 0.06	97.28 ± 0.7	41 ± 3
	2	3.09 ± 0.33	0.82 ± 0.05	96.41 ± 0.4	42 ± 1
	3	3.08 ± 0.26	0.83 ± 0.03	96.20 ± 0.32	43 ± 1
1:1	0	3.13 ± 0.05	0.64 ± 0.01	98.20 ± 0.11	23 ± 1
	1	3.11 ± 0.12	0.65 ± 0.15	97.95 ± 0.17	24 ± 2
	2	3.10 ± 0.22	0.67 ± 0.04	97.90 ± 0.20	25 ± 1
	3	3.08 ± 0.34	0.68 ± 0.06	97.86 ± 0.36	26 ± 3
1:2.5	0	3.14 ± 0.09	0.59 ± 0.03	98.75 ± 1.0	18 ± 3
	1	3.13 ± 0.92	0.60 ± 0.08	98.40 ± 0.71	19 ± 2
	2	3.11 ± 0.21	0.62 ± 0.15	97.33 ± 0.23	21 ± 4
	3	3.09 ± 0.52	0.62 ± 0.06	97.04 ± 0.87	21 ± 3
1:5	0	3.11 ± 0.06	0.56 ± 0.02	99.23 ± 0.07	15 ± 2
	1	3.11 ± 0.11	0.57 ± 0.08	98.76 ± 0.10	15 ± 7
	2	3.10 ± 0.56	0.57 ± 0.19	98.11 ± 0.18	16 ± 2
	3	3.09 ± 0.41	0.58 ± 0.03	97.99 ± 0.84	17 ± 4

**Table 10:** Stability study for the prepared CMC-SiO_2_ FDTs.

Formulation Code	Time Interval (Months)	Test Parameters (CMC-SiO_2_)			
		Hardness (kg/cm^2^)	Friability (%)	Drug Content (%)	Disintegration Time (s)
CMC	0	3.09 ± 0.08	0.86 ± 0.02	97.8 ± 0.80	43 ± 2
	1	3.07 ± 0.23	0.88 ± 0.12	97.0 ± 0.54	46 ± 2
	2	3.04 ± 0.78	0.90 ± 0.19	96.27 ± 0.67	48 ± 3
	3	3.00 ± 0.12	0.93 ± 0.23	95.28 ± 0.45	48 ± 2
1:1	0	3.11 ± 0.03	0.80 ± 0.03	98.66 ± 0.30	20 ± 3
	1	3.10 ± 0.88	0.82 ± 0.01	98.02 ± 0.73	22 ± 2
	2	3.08 ± 0.56	0.83 ± 0.10	97.89 ± 0.44	24 ± 3
	3	3.06 ± 0.33	0.85 ± 0.31	97.66 ± 0.26	25 ± 1
1:2.5	0	3.10 ± 0.14	0.73 ± 0.04	98.89 ± 1.00	18 ± 1
	1	3.09 ± 0.72	0.74 ± 0.11	98.55 ± 0.27	19 ± 3
	2	3.03 ± 0.19	0.76 ± 0.08	98.38 ± 0.19	21 ± 2
	3	3.00 ± 0.32	0.78 ± 0.02	97.94 ± 0.38	21 ± 2
1:5	0	3.12 ± 0.05	0.67 ± 0.02	99.35 ± 0.20	12 ± 2
	1	3.11 ± 0.03	0.68 ± 0.03	99.13 ± 0.88	13 ± 1
	2	3.10 ± 0.52	0.70 ± 0.13	98.78 ± 0.66	14 ± 3
	3	3.09 ± 0.31	0.71 ± 0.45	98.63 ± 0.17	14 ± 1

## Data Availability

Not applicable.
